# Multicommuted flow system for the determination of glucose in animal blood serum exploiting enzymatic reaction and chemiluminescence detection

**DOI:** 10.1155/S1463924603000191

**Published:** 2003

**Authors:** Cherrine K. Pires, Patrícia B. Martelli, Boaventura F. Reis, José L. F. C. Lima, Maria Lúcia M. F. S. Saraiva

**Affiliations:** 1 Centro de Energia Nuclear na Agricultura Universidade de São Paulo PO Box 96 Piracicaba SP 13400-970 Brazil; 2 Departamento de Química Universidade Federal de São Carlos São Carlos SP Brazil; 3 Departamento de Ciências Naturais Universidade Federal de São João Del Rei São João Del Rei MG Brazil; 4 REQUIMTE Departamento de Química-Física Faculdade de Farmácia Universidade do Porto Porto Portugal

## Abstract

An automatic flow procedure based on multicommutation dedicated for the determination of glucose in animal blood serum using glucose oxidase with chemiluminescence detection is described. The flow manifold consisted of a set of three-way solenoid valves assembled to implement multicommutation. A microcomputer furnished with an electronic interface and software written in Quick BASIC 4.5 controlled the manifold and performed data acquisition. Glucose oxidase was immobilized on porous silica beads (glass aminopropyl) and packed in a minicolumn (15 × 5 mm). The procedure was based on the enzymatic degradation of glucose, producing hydrogen peroxide, which oxidized luminol in the presence of hexacyanoferrate(III), causing the chemiluminescence. The system was tested by analysing a set of serum animal samples without previous treatment. Results were in agreement with those obtained with the conventional method (LABTEST Kit) at the 95% confidence level. The detection limit and variation coefficient were estimated as 12.0 mg l^−1^ (99.7% confidence level) and 3.5% (n = 20), respectively. The sampling rate was about 60 determinations h^−1^ with sample concentrations ranging from 50 to 600 mg l^−1^ glucose. The consumptions of serum sample, hexacyanoferrate(III) and luminol were 46 μl, 10.0 mg and 0.2 mg/determination, respectively.


Journal of Automated Methods & Management in Chemistry
Vol. 25, No. 5 (September–October 2003) pp. 109–114

Taylor & Francis

Taylor & Francis Group

### Multicommuted flow system for the determination of glucose in animal blood serum exploiting enzymatic reaction and chemiluminescence detection

**Cherrine K. Pires[1,2], Patrícia B. Martelli[3], Boaventura F. Reis[1]*, José L. F. C. Lima[4] and Maria Lúcia M. F. S. Saraiva[4]**

[1]*Centro de Energia Nuclear na Agricultura, Universidade de São Paulo, PO Box 96, 13400-970, Piracicaba — SP, Brazil* [2]*Departamento de Química, Universidade Federal de São Carlos, São Carlos — SP, Brazil* [3]*Departamento de Ciências Naturais, Universidade Federal de São João Del Rei, São João Del Rei — MG, Brazil and* [4]*REQUIMTE, Departamento de Química-Física, Faculdade de Farmácia, Universidade do Porto, Porto, Portugal*

*An automatic flow procedure based on multicommutation dedicated for the determination of glucose in animal blood serum using glucose oxidase with chemiluminescence detection is described. The flow manifold consisted of a set of three-way solenoid valves assembled to implement multicommutation. A microcomputer furnished with an electronic interface and software written in Quick BASIC 4.5 controlled the manifold and performed data acquisition. Glucose oxidase was immobilized on porous silica beads (glass aminopropyl) and packed in a minicolumn (15 × 5 mm). The procedure was based on the enzymatic degradation of glucose, producing hydrogen peroxide, which oxidized luminol in the presence of hexacyanoferrate(III), causing the chemiluminescence. The system was tested by analysing a set of serum animal samples without previous treatment. Results were in agreement with those obtained with the conventional method (LABTEST Kit) at the 95% confidence level. The detection limit and variation coefficient were estimated as 12.0 mg $l^{-1}$ (99.7% confidence level) and 3.5% ($n = 20$), respectively. The sampling rate was about 60 determinations $h^{-1}$ with sample concentrations ranging from 50 to 600 mg $l^{-1}$ glucose. The consumptions of serum sample, hexacyanoferrate(III) and luminol were 46 µl, 10.0 mg and 0.2 mg/determination, respectively.*

### Introduction

Glucose in animal blood serum is an important parameter in studies involving physiological processes and nutrition [1]. The glucose level in animal blood is dependent on the amount of glucose originating from dietary sources and those generated by animal metabolic processes such as glycogenolysis and gluconeogenesis [1,2], etc. Therefore, according to these authors, the glucose level can be used as a diagnostic parameter for animal health.

* To whom correspondence should be addressed.
e-mail: reis@cena.usp.br

Among the existing procedures for glucose determination, those based on enzymatic reaction are widely employed [3,4]. Generally, the procedures are based on the hydrogen peroxide produced during glucose oxidation by glucose oxidase (GOD) [5,6], with detection employing chemiluminescence [7], ultraviolet light (UV)-Vis spectrophotometry [8] or electrochemistry [9].

The chemiluminescent technique presents attractive features such as high sensibility and a wide dynamic range, which can be attained using inexpensive instruments [10,11]. These features have been exploited with flow analysis procedures, resulting in a useful combination considering the versatility due to the flow analysis techniques [12,13], and especially those based on sequential injection [14,15] and multicommutation [16,17].

The present paper intends to develop a multicommutated flow analysis procedure for glucose determination in animal blood serum based on enzymatic reaction and detection by chemiluminescence. The method selected is based on the reaction of luminol with hydrogen peroxide produced by the reaction of GOD with glucose [18]. Exploiting the advantages provided by the multicommutation process [19] regarding solution handling, it is intended to design a flow system to save reagents without sacrificing data quality and system robustness.

### Experimental

### *Apparatus*

The equipment set-up consisted of a 700 plus Femto spectrophotometer furnished with a home-made flat flow cell (80 $mm^2$ surface, 1.0 mm optical path length) machined in acrylic [17]; a 586 microcomputer equipped with a PCL-711S electronic interface card (Advantech Corp.); an IPC4 Ismatec peristaltic pump equipped with Tygon pumping tubes; a 12 V regulated power supply to drive the solenoid valves; a home-made electronic interface to match the voltage and current intensity required to drive the solenoid valves [19]; a minicolumn (10 mm length, 3 mm i.d.) to pack the glass beads with immobilized enzyme; joint devices machined in acrylic; and mixing coils and flow lines of polyethylene tubing (0.8 mm i.d.).

A standard UV-Vis spectrophotometer was used as a chemiluminescence detector, and for this, the light source was turned off. The spectrophotometer was set to work in the transmittance mode. The chemiluminescence flow cell was installed in the spectrophotometer about 2 mm from the photodetector inlet slit.

*Reagent solutions*

All solutions were prepared with analytical-grade chemicals. Freshly purified water presenting a conductivity of less than 0.1 μS $cm^{-1}$ was used throughout. The solutions, including samples and standards, were stored in polyethylene bottles.

A 2000 mg $l^{-1}$ glucose stock solution was prepared by dissolving sugar (Sigma Chemical Co.) in water. Reference solutions of 0.0, 50.0, 100.0, 200.0, 400.0 and 600.0 mg $l^{-1}$ glucose were prepared in water by appropriate dilution from the stock solution. These solutions were stored in a refrigerator at 5°C, and equilibrated to laboratory temperature (25°C) before use.

A 2.5 mmol 5-amino-2,3-dihydro-1,4-phthalazinedione (luminol) solution was prepared by dissolving 0.0443 g salt (Sigma) in 100 ml 0.2 mol $l^{-1}$ $K_2CO_3$ solution with the pH adjusted to 10.5 with an HCl solution.

A 0.1 mol $l^{-1}$ $K_3Fe(CN)_6$ solution was prepared daily by dissolving 3.29 g salt (Merck) in 100 ml water.

Phosphate buffer solutions 0.1 mol $l^{-1}$, pH 6.0 and 7.5, were prepared by dissolving 13.6 g $KH_2PO_4$ (Merck) in water. pHs were adjusted with a 0.1 mol $l^{-1}$ NaOH solution and volumes were made up to 1000 ml with water.

The chromogenic solution was prepared by dissolving 17 mg phenol salt in 25 ml tris(hydroxymethyl)-aminomethan solution buffer (pH 6.5) and 4 mg 4-aminoantipyrine in 50 ml of the same buffer solution. Before use, they were mixed. The 150 mmol $l^{-1}$ tris(hydroxymethyl)-aminomethan buffer solution was prepared by dissolving 4.54 g salt in water and adjusting the pH to 6.5 using a 0.2 mol $l^{-1}$ HCl solution completing the volume to 250 ml with water.

A 200 U peroxidase enzyme solution was prepared by dissolving 1 mg peroxidase in 5 ml phosphate buffer solution (pH 7.5).

A colorimetric glucose enzymatic assay, purchased from LABTEST Diagnostic (Belo Horizonte, MG, Brazil) was used to compare results.

*Enzyme immobilization*

GOD was immobilized on aminopropyl (Sigma) porous silica beads of 200–400 mesh and a pore size of 170 Å according to Matsumoto *et al.* [9]. About 0.2 g aminopropyl beads were washed first with distilled water and then with the 0.2 mol $l^{-1}$ carbonate buffer solution (pH 10.0). Afterwards, the beads were added to 20 ml 0.2 mol $l^{-1}$ carbonate solution (pH 10.0) plus 2.5 ml 5% (v/v) glutaraldehyde and stirred for 2 h while the temperature was maintained at 25°C. Afterwards, the solution was filtered and washed sequentially with both water and a 0.1 mol $l^{-1}$ phosphate buffer solution (pH 6.0).

Glucose oxidase enzyme (4.2 mg, 150 U) from *Aspergillus niger* (G-7016, Sigma) was placed in a beaker and dissolved with 20 ml 0.1 mol $l^{-1}$ phosphate buffer. The aminopropyl beads were added to this solution, which was maintained for 60 min in an ice bath at 5°C with slow stirring. Afterwards, it was filtered and conditioned in 10 ml 0.2 mg cyanoborohydride solution for 2 h, maintaining the temperature at 5°C, followed by filtration and washing with water and phosphate buffer solution. The beads were conditioned in a 0.2 mol $l^{-1}$ glycine solution medium (20 ml) for 2 h at 25°C. After this step, they were filtered and washed with phosphate buffer and inserted into the minicolumn using a syringe without a needle. When not in use, the column was maintained in a 0.1 mol $l^{-1}$ phosphate buffer solution (pH 7.5) refrigerated at 5°C.

The assay described above was repeated using intervals of 120 and 180 min.

Aiming to verify the efficiency of the immobilizing procedure, batch experiments were carried out using enzymatic solutions before and after the immobilization step. This was done by mixing 200 μl GOD solution, 200 μl peroxidase enzyme solution, 1400 μl chromogenic reagent solution and 1400 μl 300 mg $l^{-1}$ glucose solution. After a delay of 5 min, the absorbance of the product was measured at 505 nm.

*Blood sampling and conditioning*

Blood samples were collected and processed to obtain the serum as established in the literature [20,21]. This was done by puncturing the tail or jugular vein of the animals with a syringe needle of calibre 18 to prevent haemolysis. The blood was transferred to a glass tube (10 ml) previously sterilized under vacuum and centrifuged at 2500 rpm for 12 min. After this step, it was maintained at rest for 24 h at room temperature (25°C) to permit the blood clotting and serum separation. A 4-ml aliquot of serum (supernatant) was transferred to another tube that was centrifuged for 10 min to improve separation. The samples were maintained in a freezer at −4°C and before analysis they were equilibrated to room temperature.

*Flow system and procedure*

The flow network was designed to implement the multicommutation approach. The manifold is shown in figure 1. All valves are switched off and the carrier stream (C) flows through the enzymatic column and the reaction coil (B) towards the detector (D), while the sample (S) and reagent solutions ($R_1$, $R_2$) are pumped towards the storage vessels.

Because the spectrophotometer was set to work in the transmittance mode with the light source switched off, it was adjusted to 100% using a 600 mg $l^{-1}$ glucose solution at pH 7.5 and switching on $V_1$, $V_2$, $V_3$ and $V_4$ for a time of 3 min that was enough to obtain a stable measurement.

The analytical process started when the microcomputer running the software sent a sequence of electric pulses through the digital output of the PCL711S interface card to switch valves $V_1$, $V_2$, $V_3$ and $V_4$ on/off as indicated in the valves' timing course (figure 1). In the first step, the carrier solution was directed to its storage vessel and sample serum was pumped through the enzymatic

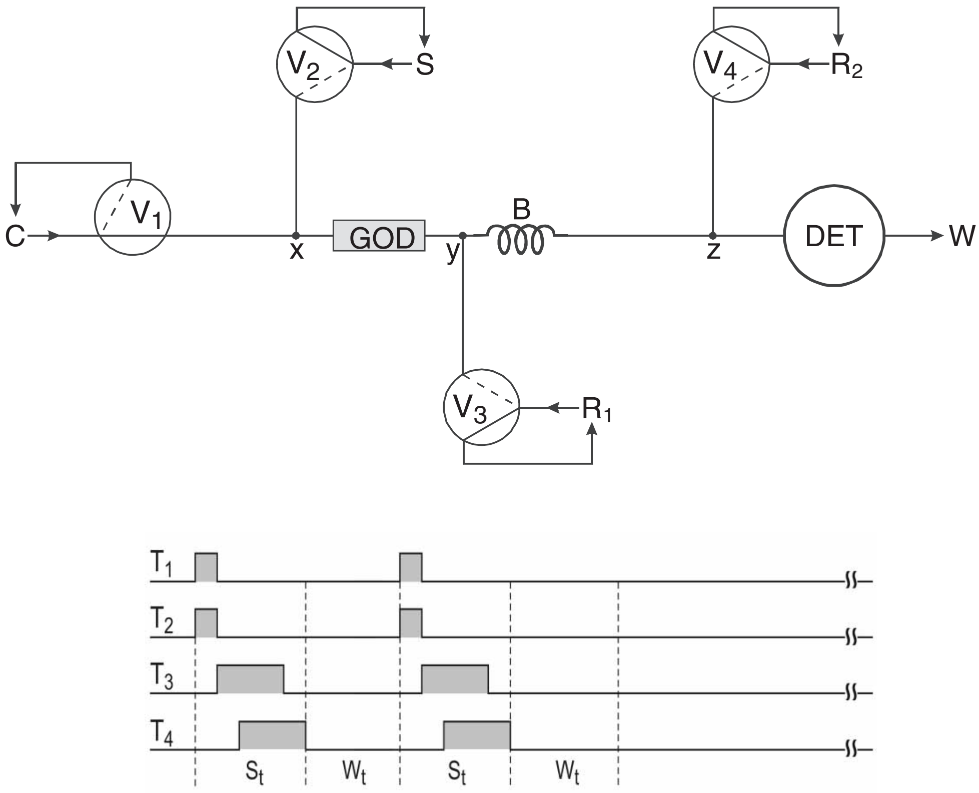

*Figure 1. Flow diagram of the system. $V_1$, $V_2$, $V_3$ and $V_4$, three-way solenoid valves; S, sample; C, carrier stream, phosphate solution at pH 7.5, flow rate at 35 μl $s^{-1}$; $R_1$, luminol solution, 2.5 mmol $l^{-1}$ flow rate at 20 μl $s^{-1}$ pH 10.5; $R_2$, hexacyanoferrate (III) solution, 0.1 mol $l^{-1}$ flow rate at 20 μl $s^{-1}$; x, y, z, joint devices; GOD, column with the immobilized enzyme; B, coiled reactor, length 75 cm, 0.8 mm i.d.; DET, chemiluminescence detector; W, waste; solid and dashed lines into the valves' symbol indicate the flow pathways when the valve is on or off, respectively. Arrows indicate the flow direction. $T_1$, $T_2$, $T_3$ and $T_4$, timing course of valves $V_1$, $V_2$, $V_3$ and $V_4$, respectively; the shadow surface beneath the valves' timing courses lines indicates that the corresponding valve was switched on.*

column (GOD) where glucose oxidation occurred. When valves $V_1$ and $V_2$ were switched off, the carrier solution flowed again through the column and reaction coil towards the detector. As indicated in the valves' timing course, in the next steps valves $V_3$ and $V_4$ were switched on to add to the sample zone the luminol and the catalyzing solutions ($R_1$, $R_2$), to allow reaction development. In these conditions, light emission due to chemiluminescence occurred within the flow cell.

The carrier fluid (C), luminol ($R_1$) and hexacyanoferrate ($R_2$) solutions and serum sample (S) were pumped at flow rates of 35, 20, 20 and 23 μl $s^{-1}$, respectively. A set of experiments to define the volumes of the aliquots of sample and of reagents solutions were carried out by varying the time intervals to switch on/off $V_1$ and $V_2$ from 1 to 4 s for the sample introduction and from 6 to 22 s for reagents $R_1$ and $R_2$. A set of glucose standard solutions ranging from 50 to 600 mg $l^{-1}$ was used throughout.

Since pH and temperature could affect the enzymatic reaction, the experiments described before were carried out using as the carrier stream a phosphate buffer solution at pH 7.5, and the temperature of the laboratory was maintained at 25°C. On the other hand, the luminol solution was prepared in buffered medium with pH adjusted to 10.5, which is the recommended value for the chemiluminescence reaction [17]. After these steps, experiments varying the pH from 6.0 to 8.5 and the temperature from 18 to 50°C were performed.

The described assays were done using luminol and hexacyanoferrate solutions with concentrations of 2.5 mmol $l^{-1}$ and 0.1 mol $l^{-1}$, respectively. Thus, to find the best condition, experiments were also carried out using concentrations ranging from 0.05 to 0.5 mol $l^{-1}$ hexacyanoferrate solutions and 0.5–4.5 mmol $l^{-1}$ luminol solutions.

Intending to demonstrate the feasibility of the proposed system, a set of serum samples was analysed without any prior treatment. The main parameters considered were precision, accuracy, reagent consumption and sampling throughput. To allow comparison, the serum samples were also analysed employing a conventional method [22].

### Results and discussion

### *Enzyme immobilization*

To find the best time to end the immobilization procedure, the absorbance of the enzymatic solutions was measured before and during the immobilization step. The initial absorbance was 0.41, and it decreased to 0.08 after 60 min. Since increasing the immobilization time up to 180 min gave no significant variation, a time of 60 min was chosen for the subsequent assays.

*Effects of sample and reagent volumes*

The flow rates of serum sample (S), luminol ($R_1$) and hexacyanoferrate(III) ($R_2$) solutions were fixed at 23, 20 and 20 μl $s^{-1}$, respectively (figure 1). The effects of sample and reagent volumes on the analytical signal were investigated by varying the time intervals to switch on valves $V_1$, $V_2$, $V_3$ and $V_4$. The volumes are summarized in table 1. Under these conditions, the sample volume that flowed through the enzymatic column during the insertion step varied from 23 to 92 μl. The maximum signal was recorded when the volumes of the inserted aliquots were 46, 360 and 320 μl for sample, luminol and hexacyanoferrate solutions, respectively. Therefore, these values were selected for further experiments. Therefore, the time intervals settled to switch on valves $V_1$, $V_2$, $V_3$ and $V_4$ were 2.0, 2.0, 18.0 and 16.0 s, respectively.

*Table 1. Effect of the sample and reagent volumes on the signal.*

| Sample volume (μl) | Reagent $R_1$ volume (μl) | Reagent $R_2$ volume (μl) | Signal (mV) |
|---|---|---|---|
| 23 | 240 | 120 | 10.6 |
| 46 | 240 | 120 | 16.8 |
| 70 | 240 | 120 | 18.2 |
| 93 | 240 | 120 | 18.0 |
| 46 | 120 | 120 | 12.6 |
| 46 | 240 | 120 | 16.8 |
| 46 | 360 | 120 | 21.2 |
| 46 | 440 | 120 | 17.0 |
| 46 | 360 | 120 | 21.2 |
| 46 | 360 | 200 | 24.2 |
| 46 | 360 | 320 | 30.6 |
| 46 | 360 | 440 | 28.4 |

Sample = 400 mg $l^{-1}$ glucose standard solution.

$R_1$ = 2.5 mmol $l^{-1}$ luminol solution; $R_2$ = 0.1 mol $l^{-1}$ hexacyanoferrate(III).

*Table 2. Effect of temperature and pH.*

| Temperature (°C) | pH | Signal (mV) |
|---|---|---|
| 18 | 7.0 | 25.0 ± 0.2 |
| 25 | 7.0 | 28.2 ± 0.1 |
| 30 | 7.0 | 31.0 ± 0.3 |
| 40 | 7.0 | 30.4 ± 0.3 |
| 50 | 7.0 | 30.2 ± 0.2 |
| 25 | 6.0 | 20.2 ± 0.1 |
| 25 | 6.5 | 22.6 ± 0.2 |
| 25 | 7.0 | 28.2 ± 0.1 |
| 25 | 7.5 | 44.8 ± 0.3 |
| 25 | 8.0 | 40.6 ± 0.1 |
| 25 | 8.5 | 40.2 ± 0.2 |

Results are the mean of three consecutive measurements.

*Effects of temperature and pH*

The enzymatic reaction can be affected by temperature and the pH of the medium. Therefore, to establish the best operational conditions, experiments were carried out varying these parameters. The results are shown in table 2. From these data, one can deduce that in the assayed temperature range, the signal increased about 20%. On the other hand, the pH of the carrier solution presented a remarkable effect showing a maximum at pH 7.5. This value was maintained for the additional experiments. Considering that the signal increasing was not significant when 30°C was employed, the experiments were carried out maintaining the temperature of the laboratory at 25°C, thus avoiding the use of a water bath with controlled temperature thus allowing a significant simplification of the equipment.

*Effect of reagent concentration*

Since the volumes of the aliquots of the reagent solutions were settled, additional experiments were performed to optimize their concentrations. The luminol concentration was changed from 0.5 to 4.5 mmol $l^{-1}$ and the generated signal increased to 2.5 mmol $l^{-1}$, thus maintaining a constant at higher values.

Similar experiments were performed to verify the catalyzing effect of the hexacyanoferrate(III) concentration, yielding the results shown in figure 2. The signal amplification was remarkable up to a 0.3 mol $l^{-1}$ hexacyanoferrate(III) concentration. The decrease observed when the reagent concentration was higher than this value was in accordance with results presented elsewhere [18]. The 0.1 mol $l^{-1}$ solution was chosen considering that the sensibility presented with this concentration was sufficient to analyse the blood serum samples.

*Sample analysis*

Considering the results discussed above, the best operational conditions (summarized in table 3) were supplied to a microcomputer when the control software was run.

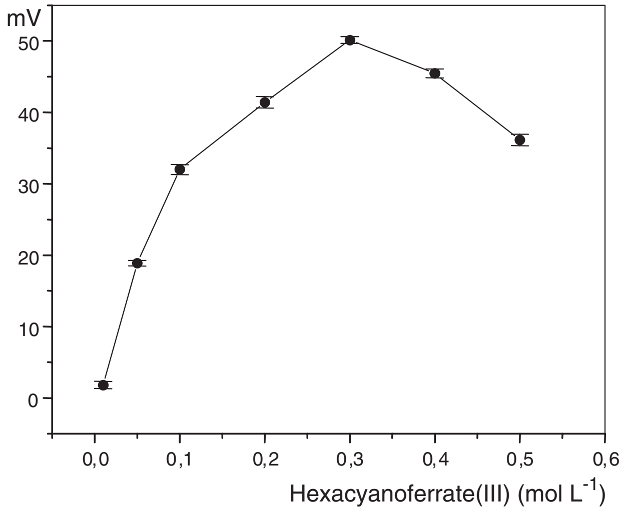

*Figure 2. Effect of hexacyanoferrate(III) concentration on the signal. System parameters were the same as those for figure 1.*

*Table 3. Operational condition of the system.*

| Step | Parameters | Valve switched on time (s) |
|---|---|---|
| Spectrophotometer calibration* | Flow rates ($\mu l\,s^{-1}$):<br>carrier = 35, sample = 20<br>luminol = 20<br>hexacyanoferrate(III) = 23 | $V_1$, $V_2$, $V_3$, $V_4$ (180) |
| Sample inserting | Sample solution volume (40 μl) | $V_1$ and $V_2$ (2.0) |
| Reagent inserting | Luminol volume (360 μl) | $V_3$ (18) |
| Reagent inserting | Hexacyanoferrate(III) solution volume (320 μl) | $V_4$ (16) |
| Washing | Carrier solution volume (525 μl) | all valves off (15) |

* Transmittance adjusting.

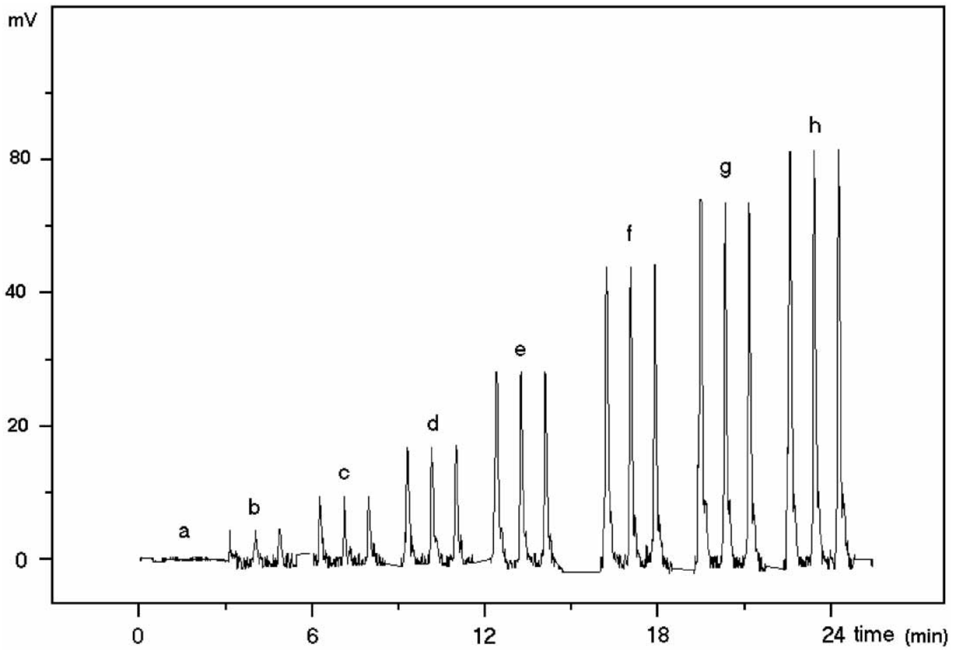

*Figure 3. Signal records of the reference solutions containing (a) br, (b) 50, (c) 100, (d) 200, (e) 300, (f) 400, (g) 500 and (h) 600 mg $l^{-1}$ glucose.*

After establishing the operational conditions, a set of animal blood serum was analysed yielding the records showed in figure 3. To validate the developed procedure, glucose was also determined using a manual procedure [22]. The results obtained using both procedures are summarized in table 4. When comparing these results by applying a paired *t*-test, no significant difference at the 95% confidence level was observed. From figure 3, it can be deduced that a sampling rate of 60 determinations $h^{-1}$ was achieved.

*Table 4. Comparison of results.*

| Sample | Proposed system ($mg\,l^{-1}$) | LABTEST Kit ($mg\,l^{-1}$) |
|---|---|---|
| 1 | 202.6 ± 7 | 198.9 ± 17 |
| 2 | 189.9 ± 3 | 186.1 ± 15 |
| 3 | 134.8 ± 4 | 138.4 ± 8 |
| 4 | 131.6 ± 1 | 135.3 ± 0 |
| 5 | 131.7 ± 25 | 117 ± 21 |

Results are the mean of three consecutive measurements ($mg\,l^{-1}$).

*Analytical figures of merit*

In the best operational conditions (summarized in table 3) when processing a set of standard solutions presenting up to 600 mg $l^{-1}$ glucose, a linear response described by the equation: signal (mV) = (2.4852 ± 0.6888) + (0.1473 ± 0.0025) mg $l^{-1}$ glucose was obtained. The blank value was 2.3957 ± 0.5987 ($n = 10$) and the limit of detection of 12 mg $l^{-1}$ glucose was estimated as three times the standard deviation of blank signal measurements divided by the slop of the calibration line.

Other profitable characteristics such as a relative standard deviation of 3.5% ($n = 20$) using a typical sample presenting 190.3 mg $l^{-1}$ glucose, a low reagent consumption of 10.0 mg hexacyanoferrate(III) and 0.2 mg luminol/determination were also observed. Furthermore, maintaining the established operational conditions, the immobilized enzyme could be used for 60 days permitting that 1200 determinations were performed.

### Conclusions

The proposed system is stable and easy to operate. A similar set of glucose standard solutions was run during four consecutive days and no significant differences in linearity and precision were observed.

The sampling rate of 60 determinations $h^{-1}$ was higher than those found in the literature, while the relative standard deviation of 3.5% ($n = 20$) was similar [12,13], confirming that laboratory productivity could be improved without sacrificing the precision of the results.

Reagent (luminol) consumption and waste generation were lower than those of the cited literature [18,23,24]. These features could be considered as an improvement provided by the multicommutation approach.

Admitting a decrease in the signal of about 20%, the immobilized enzyme allowed 1200 determinations of glucose. Note that after four weeks, the decrease in the signal during a workday was lower than 2%.

The ability of the multicommutation approach to handle tiny sample volumes permitted serum samples to be analysed without prior dilution or an additional cleaning step. This feature can be also considered as an advantage over previous methodologies.

### Acknowledgements

The authors are grateful to the Laboratory of Animal Nutrition (CENA/USP) for providing the blood samples, and CAPES/GRICES, CNPq/PRONEX and FAPESP for the financial support.

### References

1. Mascini, M. and Palleschi, G., *Anal. Chim. Acta*, **145** (1983), 213.
2. Bhagavam, N. V., *Biochemistry: A Comprehensive Review* (Philadelphia: J. B. Lippincott, 1974).
3. Guilbault, G. G., Brignac, Jr., P. and Zimmer, M., *Anal. Chem.*, **38** (1966), 190.
4. Maason, M. and Townshend, A., *Anal. Chim. Acta*, **166** (1984), 111.
5. Milardovic, S., Kruhak, I., Ivekovic, D., Rumenjak, V., Tkalcec, M. and Grabaric, B. S., *Anal. Chim. Acta*, **350** (1997), 91.
6. Harms, D., Meyer, J., Westerheide, L., Krebs, B. and Karst, U., *Anal. Chim. Acta*, **401** (1999), 83.
7. Zhou, Y., Nagaoka, T., Li, F. and Zhu, G., *Talanta*, **48** (1999), 461.
8. Maeuchihara, N., Nakano, S. and Kawashima, T., *Anal. Sci.*, **17** (2001), 255.
9. Matsumoto, K., Kamikado, H., Matsubara, H. and Osajima, Y., *Anal. Chem.*, **60** (1988), 147.
10. Koerner, C. A. and Nieman, T. A., *Anal. Chem.*, **58** (1986), 116.
11. Paul, D. B., *Talanta*, **25** (1978), 377.
12. Laespada, M. E. F., Pavón, J. L. P. and Cordero, B. M., *Anal. Chim. Acta*, **327** (1996), 253.
13. Qin, W., Zhang, Z., Li, B. and Liu, S., *Anal. Chim. Acta*, **372** (1998), 357.
14. Tucker, D. J., Toivola, B., Pollema, C. H., Ruzicka, J. and Christian, G. D., *Analyst*, **119** (1994), 975.
15. Pasekova, H., Polasek, M., Cigarro, J. F. and Dolejsova, J., *Anal. Chim. Acta*, **438** (2001), 165.
16. Fernandes, R. N., Reis, B. F. and Campos, L. F. P., *J. Autom. Meth. Mgmt. Chem.*, **25** (2003), 1.
17. Martelli, P. B., Reis, B. F., Araújo, A. N. and Montenegro, M. C. B. S. M., *Talanta*, **54** (2001), 879.
18. Ridder, C., Hansen, E. H. and Ruzicka, J., *Anal. Lett.*, **15** (1982), 1751.
19. Reis, B. F., Giné, M. F., Zagatto, E. A. G., Lima, J. L. F. C. and Lapa, R. A. S., *Anal. Chim. Acta*, **293** (1994), 129.
20. Christian, G., *Analytical Chemistry* (New York: Wiley, 1986).
21. FAO/IAEA, *Programme of Animal Production and Health: Nutritional Metabolite Kit Protocols* (Seibersdorf: Agency's Laboratories, 1993).
22. LABTEST Diagnostica, *Colorimetric Glucose Enzymatic Assay* (Belo Horizonte, 1999).
23. Hansen, E. H., Norgaard, L. and Pedersen, M., *Talanta*, **38** (1991), 275.
24. Worsfold, P. J., Farrelly, J. and Matharu, M. S., *Anal. Chim. Acta*, **164** (1984), 103.

